# Measuring Values in Environmental Research: A Test of an Environmental Portrait Value Questionnaire

**DOI:** 10.3389/fpsyg.2018.00564

**Published:** 2018-04-23

**Authors:** Thijs Bouman, Linda Steg, Henk A. L. Kiers

**Affiliations:** Department of Psychology, Faculty of Behavioural and Social Sciences, University of Groningen, Groningen, Netherlands

**Keywords:** value, Schwartz Value Survey, SVS, Portrait Value Questionnaire, PVQ, environmental behavior, environmental beliefs, value measurement

## Abstract

Four human values are considered to underlie individuals’ environmental beliefs and behaviors: biospheric (i.e., concern for environment), altruistic (i.e., concern for others), egoistic (i.e., concern for personal resources) and hedonic values (i.e., concern for pleasure and comfort). These values are typically measured with an adapted and shortened version of the Schwartz Value Survey (SVS), to which we refer as the Environmental-SVS (E-SVS). Despite being well-validated, recent research has indicated some concerns about the SVS methodology (e.g., comprehensibility, self-presentation biases) and suggested an alternative method of measuring human values: The Portrait Value Questionnaire (PVQ). However, the PVQ has not yet been adapted and applied to measure values most relevant to understand environmental beliefs and behaviors. Therefore, we tested the Environmental-PVQ (E-PVQ) – a PVQ variant of E-SVS –and compared it with the E-SVS in two studies. Our findings provide strong support for the validity and reliability of both the E-SVS and E-PVQ. In addition, we find that respondents slightly preferred the E-PVQ over the E-SVS (Study 1). In general, both scales correlate similarly to environmental self-identity (Study 1), energy behaviors (Studies 1 and 2), pro-environmental personal norms, climate change beliefs and policy support (Study 2). Accordingly, both methodologies show highly similar results and seem well-suited for measuring human values underlying environmental behaviors and beliefs.

## Introduction

Haltering global warming is one of the main challenges of our times and probably one of the biggest global challenges mankind ever faced. Despite most individuals being aware of the problem and the obvious role mankind has in it, many people still not engage in sustainable behavior consistently (Dietz et al., 2009; Steg et al., 2015; Stern et al., 2016; Leiserowitz et al., 2017). One of the main goals within environmental research is to understand and predict differences in environmental beliefs and behaviors and find ways to motivate people to act more pro-environmentally.

A key determinant of a wide range of environmental beliefs and behaviors are personal values (Stern et al., 1998; Schultz and Zelezny, 1999; Dietz et al., 2005; De Groot and Steg, 2008; Steg et al., 2014; Hornsey et al., 2016). Values are general goals where people strive for in life (Schwartz, 1992). They transcend specific situations, are relatively stable over time and affect a wide range of beliefs and behaviors (Rokeach, 1973; Schwartz, 1992). Four types of values proved to be most relevant in predicting environmental beliefs and behaviors (Stern et al., 1998; De Groot and Steg, 2008; Steg et al., 2014): biospheric (i.e., valuing the environment), altruistic (i.e., valuing the welfare and wellbeing of other human beings), egoistic (i.e., valuing personal resources) and hedonic values (i.e., valuing pleasure and comfort). These values are typically measured with a validated value instrument (Stern et al., 1998; De Groot and Steg, 2008; Steg et al., 2014), which is a brief and adapted version of the Schwartz Value Survey^1^ (SVS, Schwartz, 1994; Stern et al., 1998), to which we refer as the Environmental-SVS (E-SVS).

Although the E-SVS is commonly used and well-validated, research indicated that respondents often find it difficult to answer SVS items – as we will discuss in more detail later – and that these difficulties sometimes result in deviations from the theorized value structure (Schwartz et al., 2001; Schwartz, 2003). Therefore, Schwartz et al. (2001) and Schwartz (2003) developed the Portrait Value Questionnaire (PVQ), which aims to measure the same values as the SVS in a simpler way. Environmental research might also benefit from adopting the PVQ methodology. However, since the E-SVS items slightly differ from the items included in the full SVS (Stern et al., 1998; De Groot and Steg, 2008; Steg et al., 2014), and both identify different value clusters, an adjusted version of the PVQ is needed to measure those values relevant for environmental research, in which the E-SVS items are adapted to the PVQ methodology. To our knowledge, there is no validated Environmental PVQ (E-PVQ) available yet. Accordingly, it is yet unknown whether an E-PVQ is a good – or even better – alternative for the E-SVS. Therefore, in two studies, we aim to validate an E-PVQ comprising of similar items as the E-SVS, and compare its reliability, factor structure and predictive power to the original E-SVS. Before turning to these studies, we will first in more detail explain values, their relationship with environmental beliefs and behaviors, and the SVS and PVQ methodologies.

### Personal Values

As values resemble general guiding principles in people’s lives (Durkheim, 1897; Weber, 1905; Rokeach, 1973; Schwartz and Bilsky, 1987; Maio, 2010; Schwartz, 2012), they are considered to be deep-rooted personal criteria on which thoughts and actions are, often unconsciously, based and evaluated (Feather, 1995; Schwartz, 2012). Although all individuals endorse the same values to some extent (Rokeach, 1973), individuals differ in the way they prioritize certain values over others. These differences in value priorities – for instance, valuing the environment (i.e., biospheric values) more than the gratification of personal desires (i.e., hedonic values) – determine the choices individuals eventually make. The more individuals endorse a specific value, the more strongly they are likely to think and behave in line with this value. Values mostly affect behavior indirectly, through for example environmental self-identity and personal norms (e.g., Stern et al., 1999; Poortinga et al., 2012; Van Der Werff et al., 2013a; Van Der Werff and Steg, 2015; Ruepert et al., 2016).

Values are typically organized on a circular complex, consisting of a ‘self-transcendence’ to ‘self-enhancement’ dimension and an ‘openness to change’ to ‘conservation’ dimension (Schwartz, 1994). Pro-environmental beliefs, attitudes, identities, and behaviors – such as energy saving behaviors, recycling and acceptability of environmental policies – proved to particularly relate to values belonging to the self-transcendence and self-enhancement dimension (Stern et al., 1998; Schultz and Zelezny, 1999; Stern, 2000; Nordlund and Garvill, 2002, 2003; Milfont and Gouveia, 2006; De Groot and Steg, 2007, 2008, 2009; Hansla et al., 2008; Boer and Fischer, 2013; Steg et al., 2014; Nilsson et al., 2016). Self-transcendence values make individuals focus on the interests of others and the environment (Schwartz, 1992, 2012) and are typically positively related to pro-environmental beliefs and behaviors (Stern et al., 1998; Schultz and Zelezny, 1999; Boer and Fischer, 2013; Steg et al., 2014). Conversely, self-enhancement values make individuals focus on self-interests (Schwartz, 1992, 2012) and are generally negatively related to pro-environmental beliefs and behaviors (Stern et al., 1998; Schultz and Zelezny, 1999; Steg et al., 2014).

Notably, within environmental research, two types of self-transcendence values and two types of self-enhancement values are typically differentiated (Stern et al., 1998; De Groot and Steg, 2008; Steg et al., 2014). Self-transcendence values include biospheric and altruistic values (Stern et al., 1998; De Groot and Steg, 2008; Steg et al., 2014). Although this distinction has not been made in the SVS (Schwartz, 1992), it is important to differentiate them since both values represent different motivations to think and act pro-environmentally (Stern and Dietz, 1994; Stern, 2000; De Groot and Steg, 2008). *Biospheric values* reflect a concern for the environment in itself, without a clear link to human beings. Accordingly, acting pro-environmentally would directly support this value and biospheric values are indeed, when compared to other values, most strongly and consistently related to pro-environmental beliefs and behaviors^2^. *Altruistic values* reflect a concern for the welfare and fair treatment of other human beings. As pro-environmental beliefs and behaviors oftentimes relate to positive outcomes for human beings (e.g., health benefits) or are seen as a requirement to preserve our planet for future generations, altruistic values are typically also positively related to pro-environmental beliefs and behaviors when such behaviors also benefit other people (e.g., Stern et al., 1998; De Groot and Steg, 2007; Perlaviciute and Steg, 2014). However, altruistic and biospheric values sometimes conflict, for instance when someone has to choose between donating to humanitarian or environmental organizations (De Groot and Steg, 2008), in which case strong altruistic values inhibit pro-environmental choices. Accordingly, biospheric and altruistic values relate to each other, but are conceptually and empirically distinguishable (De Groot and Steg, 2008), and both generally relate positively to pro-environmental beliefs and behaviors, unless they conflict each other (De Groot and Steg, 2008).

Self-enhancement values include egoistic (Schwartz, 1994; Stern et al., 1998; De Groot and Steg, 2008; Schwartz et al., 2012; Steg et al., 2014) and hedonic values (Schwartz, 1994; Schwartz et al., 2012; Steg et al., 2014). *Egoistic values* reflect a focus on the costs and benefits a choice has on someone’s resources, and on power or achievement. *Hedonic values* focus on attaining pleasure, positive feelings and reducing effort. Many environmental behaviors are associated with egoistic and hedonic costs as these behaviors are often considered – but not necessarily have to be (Venhoeven et al., 2013) – effortful (e.g., using public transport instead of taking the car), uncomfortable (e.g., lowering the heating) or costly (e.g., buying organic products). Accordingly, individuals who strongly endorse egoistic or hedonic values are typically less inclined to act pro-environmentally and have less strong pro-environmental beliefs. However, when pro-environmental behaviors do have egoistic or hedonic benefits, for instance when energy savings also imply saving money, and when organic produce is also more tasty, egoistic or hedonic values might positively relate to these behaviors.

In sum, individuals who strongly endorse biospheric and altruistic values typically act more pro-environmentally and hold stronger pro-environmental beliefs, whereas those who strongly endorse egoistic and hedonic values are less inclined to do and think so. Research has indicated that these relationships are consistently found for a wide range of environmental beliefs and behaviors and are observable across various cultures and countries (Dietz et al., 2005; De Groot and Steg, 2007; cf. Schultz and Zelezny, 1999; Steg and De Groot, 2012), which is why these four values are frequently used and measured within environmental research.

### Measuring Values in Different Ways

The four values relevant for environmental research are typically measured with the E-SVS (Stern et al., 1998; De Groot and Steg, 2008; Perlaviciute and Steg, 2014). The E-SVS consists of 16 items (see **Table 1**), including 14 items from the original SVS (Schwartz, 1994) and 2 additional biospheric value items because these were underrepresented in the original SVS (Stern et al., 1998). In the SVS methodology, participants are presented with a list of different values, which includes a short title (e.g., “UNITY WITH NATURE”) and a brief description (e.g., “fitting into nature”), and are asked to indicate on a 9-point scale (-1 *opposed to my values* to 0 *not important* to 7 *of supreme importance*) how important each value is as a guiding principle in their lives. In addition, respondents are typically prompted to vary their responses to ensure enough variation between their answers.

**Table 1:** Items for measuring the four values related to environmental behaviors and beliefs, based on the original Environmental-SVS (E-SVS) methodology (left; Steg et al., 2014) and the newly proposed Environmental-PVQ (E-PVQ) methodology (right).

	E-SVS; Steg et al., 2014	E-PVQ
Biospheric		
Bio1	PREVENTING POLLUTION (protecting natural resources)	It is important to [him/her] to prevent environmental pollution.
Bio2	PROTECTING THE ENVIRONMENT (preserving nature)	It is important to [him/her] to protect the environment.
Bio3	RESPECTING THE EARTH (harmony with other species)	It is important to [him/her] to respect nature.
Bio4	UNITY WITH NATURE (fitting into nature)	It is important to [him/her] to be in unity with nature.
Altruistic		
Alt1	EQUALITY (equal opportunity for all)	It is important to [him/her] that every person has equal opportunities.
Alt2	SOCIAL JUSTICE (correcting injustice, care for the weak)	It is important to [him/her] to take care of those who are worse off.
Alt3	———————	It is important to [him/her] that every person is treated justly.
Alt4	A WORLD AT PEACE (free of war and conflict)	It is important to [him/her] that there is no war or conflict.
Alt5	HELPFUL (working for the welfare of others)	It is important to [him/her] to be helpful to others^a^.
Hedonic		
Hed1	PLEASURE (gratification of desires)	It is important to [him/her] to have fun.
Hed2	ENJOYING LIFE (enjoying food, sex, leisure, etc.)	It is important to [him/her] to enjoy the life’s pleasures.
Hed3	SELF-INDULGENT (doing pleasant things)	It is important to [him/her] to do things [he/she] enjoys.
Egoistic^b^		
Ego1	SOCIAL POWER (control over others, dominance)	It is important to [him/her] to have control over others’ actions.
Ego2	AUTHORITY (the right to lead or command)	It is important to [him/her] to have authority over others.
Ego3	INFLUENTIAL (having an impact on people and events)	It is important to [him/her] to be influential.
Ego4	WEALTH (material possessions, money)	It is important to [him/her] to have money and possessions.
Ego5	AMBITIOUS (hardworking, aspiring)	It is important to [him/her] to work hard and be ambitious.

*For a direct comparison between the exact items of the SVS (Schwartz, 1992), E-SVS (Steg et al., 2014), PVQ5X – PVQ-R (Schwartz et al., 2012), PVQ-RR (Schwartz, unpublished) and E-PVQ, see Supplementary Tables S1–S4. ^a^In Study 1, this item also included “… and work for the welfare of others” at the end, which we removed after feedback from respondents. ^b^For explorative purposes, two items were added to the PVQ questionnaire “It is important to [him/her] to show [he/she] is successful” and “It is important to him/her that people recognize what he/she achieves.” Because this paper focused on the comparison between SVS and PVQ items, we decided to not include them in the current analyses to keep both scales as similar as possible.*

Although the E-SVS is well-validated (Stern et al., 1998; De Groot and Steg, 2007, 2008; Steg et al., 2014), recent research on the original SVS indicated some concerns about the SVS methodology. Some of these concerns focus on the direct way of asking respondents about their values, which might be problematic for at least three reasons (Schwartz, 2003). Firstly, respondents see most values as important and – when asked directly – find it hard to prioritize one value over another. Although Schwartz (1992, 1994) partly accounts for this by employing a non-symmetrical 9-point scale with relatively many scale points between *important* (3) and *of extreme importance* (7), many respondents may still find it hard to differentiate between the items in their scoring. This could frustrate respondents and cause invariance in their answering, which makes the data less reliable and hard to interpret. Secondly, respondents might start ruminating about how values relate to each other due to the direct way of asking, and think about their answers too much. Research has indicated that actively reflecting on ones values might change individuals’ value ratings and reduce the predictive power of these values (Maio and Olson, 1998). Lastly, the direct way of asking is also likely to make respondents focus on self-presentation. Some values might be regarded as more socially accepted than others, which makes this methodology vulnerable to self-enhancement biases (Schwartz, 2003, 2005).

In addition to these issues related to the direct way of asking, specific groups (e.g., children under 14, elderly and people who did not follow typical Western education) have difficulties with completing the (E-)SVS as they are not used to the abstract, context-free formulation of the items, which could result in measuring errors (Schwartz et al., 2001, 2012; Schwartz, 2003; Maio, 2010; Schwartz and Butenko, 2014). Furthermore, since distances between the scale points of the non-symmetrical 9-point scale are not equal to each other, the (E-)SVS methodology could be considered demanding to respondents and prone to measuring errors (e.g., respondents might fill it out as if it is a symmetrical scale because they are more used to this), which complicates statistical analyses and interpretation.

Because of the aforementioned concerns, Schwartz et al. (2012), Cieciuch et al. (2014), and Torres et al. (2016) developed and tested the PVQ, which aims to measure the same values in a less direct, easier and more respondent-friendly manner. Instead of asking about the respondents’ values directly, the PVQ uses short verbal portraits of another person – gender-matched to the participant – in which a value is described that is important to this person (e.g., “It is important to him to enjoy life’s pleasures.”). Respondents are asked to indicate the extent to which each portrayed person is like the respondent himself or herself, ranging from 1 (*not like me at all*) to 6 (*very much like me*). The wording of the PVQ value descriptions (e.g., “It is important to him to enjoy life’s pleasures”) aims to match the original SVS value description (e.g., “ENJOYING LIFE: enjoying food, sex, leisure, etc.”).

The formulation of the PVQ aims to address the concerns associated with the SVS, as respondents are more used to rate how similar others are to themselves (i.e., PVQ) than to rate the values themselves directly (i.e., SVS; Schwartz, 2003, 2012). Accordingly, the PVQ methodology aims to increase the comprehensibility of the PVQ items compared to the SVS items, making it easier for respondents to answer the questions, taking them less time to complete the questionnaire and possibly yielding more reliable indexes of individuals’ basic values (Schwartz et al., 2012). In addition, because the PVQ formulation focuses on evaluations of other persons rather than evaluations of the self, answers are expected to be less influenced by self-presentation. Respondents are less likely to think about what is socially acceptable and how the values might relate to each other (Schwartz, 2003). Furthermore, the more symmetrical scale employed by the PVQ is believed to have both practical (e.g., comprehensibility, ease-of-use) and statistical advantages over the SVS scale.

Comparisons between the SVS and PVQ indicated that both methodologies are in general reliable and successfully distinguish between values (for the exact items used within each scale, please see Supplementary Tables S1–S4). Furthermore, value scales based on both methodologies strongly relate to each other (Schwartz, 2003, 2005; Schmidt et al., 2007). Moreover, although seldom directly compared, the relationship between specific values and constructs such as age, gender, education, political orientation (Schwartz, 2003, 2005; Schmidt et al., 2007), and environmental preferences (Schmidt et al., 2007), are comparable among measures. Despite these similarities, however, the PVQ seems more successful in identifying value structures in non-Western populations (Schwartz et al., 2001) and among children (Schwartz, 2005; Cieciuch et al., 2013), which appears to be in line with the methodology’s potential advantages.

Nevertheless, there is still much unknown about the merits of each measure and whether they predict outcome variables in the same way. More specific to the environmental domain, the question remains whether an E-PVQ (i.e., the E-SVS adapted to the PVQ methodology) can replace the E-SVS as an important predictor of environmental beliefs and behaviors. As described before, the E-SVS is a shortened and adapted version of the SVS that specifically measures those values that are most relevant for understanding environmental beliefs and behaviors. Moreover, the E-SVS and SVS identify slightly different value clusters. Particularly the distinction between biospheric and altruistic values in the E-SVS is important in environmental research (Stern, 2000; De Groot and Steg, 2008), which is not reflected in the SVS (Schwartz, 1992) and not fully reflected in the PVQ (Schwartz et al., 2012).

We propose a short and adapted version of the PVQ for environmental research – the E-PVQ – in which each E-SVS item is merely rephrased to fit the PVQ methodology, making sure we retained the original content as much as possible, thereby enabling a fair comparison. We tested the reliability and validity of the E-PVQ. More specifically, we directly compare the E-PVQ with the original E-SVS in order to (1) investigate whether both scales identify the same value structure, (2) test and compare the reliability of both scales, (3) inspect the correlation between the E-PVQ and E-SVS value clusters, (4) examine each scale’s ability to predict relevant environmental outcome variables and (5) evaluate each scale’s ease-of-use (the latter only Study 1).

## Study 1

### Materials and Methods

#### Participants

An online questionnaire study was conducted in 2016. Participants were recruited through email and/or social media. In total, 53 Dutch individuals volunteered to participate in our questionnaire, of which 36 were females. The mean age was 32.4 years (*SD* = 14.01). Two respondents had missing data on the energy behavior measures and scale evaluations; accordingly, analyses involving these variables were done among the remaining 51 respondents.

#### Procedure and Measures

After agreeing on informed consent, participants could start the online questionnaire. Participants were first asked for their gender and birth year, after which they completed both value scales; the order of the value scales was randomized. Thereafter, common environmental correlates of values were presented – environmental self-identity and a variety of energy saving behaviors. Lastly, participants were asked to compare both value scales with each other on ease of use, and to review and evaluate the comprehensibility of the E-PVQ items.

##### Values

Biospheric, altruistic, hedonic and egoistic values were measured with both the E-SVS and E-PVQ. The E-SVS (see Steg et al., 2014) consisted of 16 items containing descriptions of the relevant values (see **Table 1**). For each item, participants were asked to indicate on a 9-point scale (-1 *opposed to my values* to 0 *not important* to 3 *important* to 6 *very important* to 7 *of supreme importance*) how important each value is as a guiding principle in their life. The E-PVQ consisted of 17^3,4^ items containing descriptions, which were based on the E-SVS content, of what another person (gender matched) thought was very important in life (see **Table 1**). Participants were asked to respond on a 7-point^5^ scale (1 *not like me at all* to 7 *very much like me*) how much the person in the description was similar to themselves. For both scales, participants were asked to differentiate as much as possible between the items, to ensure that participants distinguished between the importance of the different values. For both scales separately, we did reliability analyses and used confirmatory factor analysis to inspect whether we could confirm the theorized value structure; after which we computed a composite score for each value cluster (i.e., biospheric, altruistic, hedonic, and egoistic values) by calculating the mean of the respective items (for descriptive statistics and reliability, see **Table 2**).

**Table 2:** Corrected correlations between value items and value clusters for both E-SVS and E-PVQ via oblique multiple group method, and the correlation between the E-SVS and corresponding E-PVQ item.

	Value cluster								
	E-SVS				E-PVQ				
	Bio	Alt	Hed	Ego	Bio	Alt	Hed	Ego	*r*
**Biospheric**	**Cronbach’s alpha = 0.875**				**Cronbach’s alpha = 0.877**				**0.79**

Pollution	**0.75**	0.35	-0.12	0.10	**0.76**	0.35	-0.21	-0.11	0.72
Protection	**0.83**	0.35	-0.16	-0.05	**0.81**	0.18	-0.07	-0.04	0.66
Respect	**0.73**	0.41	-0.19	0.03	**0.76**	0.20	-0.12	0.06	0.51
Unity	**0.63**	0.31	-0.21	-0.01	**0.64**	0.20	-0.09	-0.02	0.68

**Altruistic**	**Cronbach’s alpha = 0.630**				**Cronbach’s alpha = 0.681**				**0.66**

Equal	0.26	**0.54**	-0.02	0.01	0.03	**0.48**	0.05	-0.07	0.54
Social justice	0.23	**0.59**	-0.18	-0.03	0.18	**0.50**	-0.06	-0.01	0.57
Taking care	–	**–**	–	–	0.40	**0.58**	-0.17	-0.13	0.64
Peace	0.48	**0.18**	-0.34	-0.07	0.15	**0.21**	-0.10	-0.14	0.48
Helpful	0.14	**0.41**	0.06	-0.01	0.10	**0.47**	-0.03	0.11	0.70

**Hedonic**	**Cronbach’s alpha = 0.781**				**Cronbach’s alpha = 0.778**				**0.71**

Pleasure/fun	-0.17	-0.19	**0.58**	0.46	-0.13	-0.13	**0.71**	0.33	0.66
Enjoying life	-0.20	-0.22	**0.72**	0.33	-0.10	-0.03	**0.61**	0.07	0.36
Self-indulgent	-0.13	-0.06	**0.59**	0.12	-0.14	-0.11	**0.55**	0.37	0.49

**Egoistic**	**Cronbach’s alpha = 0.741**				**Cronbach’s alpha = 0.708**				**0.77**

Social power	-0.01	-0.27	0.19	**0.47**	0.01	-0.03	0.14	**0.47**	0.46
Authority	0.07	0.01	0.18	**0.69**	0.18	-0.07	0.17	**0.49**	0.66
Influential	0.18	0.22	0.05	**0.54**	0.05	-0.08	0.11	**0.55**	0.31
Wealth	-0.09	-0.11	0.16	**0.52**	-0.22	-0.15	0.18	**0.41**	0.74
Ambitious	-0.06	-0.03	0.58	**0.37**	-0.06	0.02	0.37	**0.45**	0.86

*M*	4.17	4.94	5.00	2.06	4.52	5.48	5.87	3.33	
*SD*^a^	1.39	1.02	1.15	1.16	1.12	0.77	0.81	0.91	

*Boldfaced correlation coefficients indicate the corrected item-total correlations between an item and the value cluster to which the item belongs. Correlation coefficients larger than 0.24 had one-sided p-values below 0.05. ^a^We report untransformed SDs. For direct comparisons, one should adjust for the measuring scale used, which could be done by dividing the SDs of the E-SVS by 9 and multiplying them by 7. This results in SDs of 1.13, 0.83, 1.05, and 0.85, respectively, which are comparable to the SDs observed for the E-PVQ.*

##### Environmental self-identity

Environmental self-identity reflects the extent to which someone perceives oneself as the type of person who acts environmentally friendly (Whitmarsh et al., 2010; Van Der Werff et al., 2013a; Lacasse, 2016; Uhl et al., 2016). Although related to biospheric values, it is conceptually different as it reflects how individuals see themselves, which does not necessarily reflect what they value (Van Der Werff et al., 2013b). We measured environmental self-identity with three items (e.g., I am the type of person who acts environmentally friendly, see Van Der Werff et al., 2013b), which participants had to rate on a 7-point scale (1 *totally disagree* to 7 *totally agree*). We computed a composite score by calculating the mean of all items (α = 0.89, *M* = 4.15, *SD* = 1.10).

##### Energy behaviors

We selected 11 energy behaviors that are expected to correlate with values based on earlier research (Thøgersen and Ölander, 2003; Thøgersen, 2004; Abrahamse et al., 2007; Whitmarsh et al., 2010; Lanzini and Thøgersen, 2014; Steinhorst et al., 2015). Four items were related to energy wasting behaviors and asked about the relative frequency of (1) leaving lights on when no one is in the room, (2) leaving appliances on stand-by, (3) doing laundry with the machine not fully loaded and (4) boiling more water than necessary (7-point scale, 1 *never* to 7 *always*). Four items were related to energy saving behaviors, asking about the relative frequency of (1) doing laundry on cold temperature, (2) lowering temperature when nobody is at home, (3) lowering temperature at night and (4) lowering temperature 30 min before going out or going to bed (7-point scale, 1 *never* to 7 *always*). Lastly, we asked about their average temperature in the living room when heating is on (degrees Celsius), how frequent participants typically shower and/or bath a week, and how long an average shower takes (in minutes). Since previous research indicated that these energy behaviors often weakly relate to each other and differ in their relationship with values (Thøgersen and Ölander, 2003; Thøgersen, 2004; Abrahamse et al., 2007; Whitmarsh et al., 2010; Lanzini and Thøgersen, 2014; Steinhorst et al., 2015), we analyzed the behaviors separately rather than focusing on a composite score. By doing so, we could test whether similar results were found for a range of energy-behavior variables.

##### Comparison E-SVS and E-PVQ

We asked respondents to compare the E-SVS and E-PVQ on their ease-of-use, clarity and comprehensibility. On each item, participants could indicate whether they either preferred the E-PVQ (1), the E-SVS (-1) or no preference for one over the other (0).

##### Comprehensibility of E-PVQ

Lastly, as the E-PVQ was a newly developed tool, participants were asked to evaluate and review the clarity of the E-PVQ specifically. Participants scored the introduction and each of the 17 E-PVQ items on a 5-point scale, ranging from 1 *very incomprehensible* to 5 *very comprehensible*, and were given the opportunity to elaborate on their score.

### Results

Descriptive statistics on the comprehensibility of the E-PVQ showed that the introduction text (*M* = 4.20, *SD* = 0.83) and all items were easy to understand (*Ms* = 4.16 to 4.71, *SDs* = 0.50 to 1.03). In addition, respondents slightly preferred the E-PVQ items over the E-SVS items. On ease, 22 subjects preferred the E-PVQ, 10 the E-SVS, while 19 had no preference; for clarity 21 subjects preferred the E-PVQ, 14 the E-SVS, and 16 had no preference; and for comprehensibility 22 subjects preferred the E-PVQ, 9 the E-SVS, and 20 had no preference. Accordingly, the mean scores on the -1 (preference for E-SVS), 0 (no preference), 1 (preference for E-PVQ) preference scales were *M* = 0.24, *SD* = 0.76 for ease, *M* = 0.14, *SD* = 0.83 for clarity, and *M* = 0.25, *SD* = 0.74 for comprehensibility. Despite the fact that distributions are not normal, the means can still be expected to be distributed sufficiently normally to use *t*-statistics, and compute the associated 95% confidence intervals, which were as follows: 0.02 to 0.45 for ease, -0.09 to 0.37 for clarity, and 0.05 to 0.46 for comprehensibility.

#### Verifying Value Clusters

The oblique multiple group method (Guttman, 1952; Nunnally, 1978; Stuive, 2007) type of confirmatory factor analysis was used to verify whether the items grouped on the corresponding predefined value clusters. We choose this type of confirmatory factor analysis because research has indicated that its results are similar to the more commonly used structural equation modeling (SEM), but that the OMG method is much easier and less ambiguous to interpret (Stuive, 2007; Stuive et al., 2009). Moreover, in previous research OMG performed similar or better than SEM in identifying E-SVS value clusters (De Groot and Steg, 2007; Stuive, 2007) and is regarded highly insightful when inspecting the items’ relationships with the value clusters. Following this approach, we first calculated the composite value scales (i.e., mean score of values belonging to the same value cluster) based on theoretical grounds. Next, for each value item we calculated its correlation with each of the composite value scales – the correlation between an item and the scale to which it was supposed to belong being corrected for “self-correlation.” Finally, we verified whether the corrected correlation between an item and the scale to which it was supposed to belong was stronger than its correlation with the other scales, which indicates support for the theorized value structure (Nunnally, 1978).

**Table 2** shows that for the E-SVS all but two items correlated strongest with the value scale with which it was supposed to be associated. The item “a world at peace” did not significantly correlate with the altruistic value scale to which it theoretically belongs; instead, it correlated most strongly with the biospheric value scale. The item “ambitious” did significantly correlate with the egoistic value scale to which it theoretically belongs, but correlated slightly stronger with the hedonic value scale. For the E-PVQ (see **Table 2**), all items correlated strongest with the value scale to which the item theoretically belonged. However, like the E-SVS, the item focusing on peace (i.e., “… no war or conflict”) did only weakly correlate to the altruistic value scale.

#### Multidimensional Scaling

To further inspect how the value items of the E-SVS and E-PVQ cluster together we performed a multidimensional scaling (MDS) of the variables, based on the Euclidean distances between the scores on the variables. For this purpose, we used the PROXSCAL program in SPSS, employing 20 random starting configurations as well as the classical Torgerson starting configuration, and set the convergence values at 0.0001, and fixed the maximum number of iterations to 100. We only report two dimensional solutions, as these portray the structure well enough [dispersion accounted for (DAF) around 95%].

**FIGURE 1:**
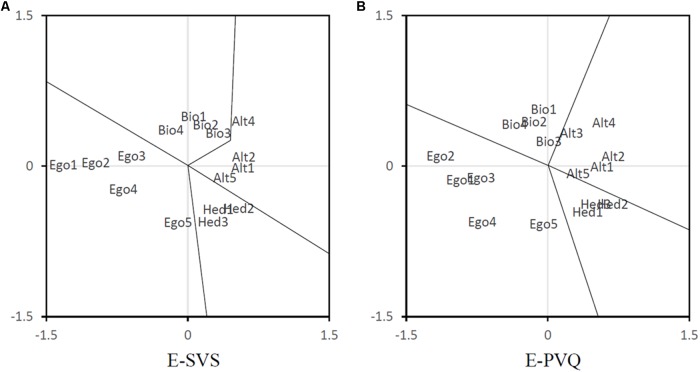
Two-dimensional multidimensional scaling for the **(A)** E-SVS and **(B)** E-PVQ tested in Study 1. Item abbreviations (e.g., Bio1) correspond to the ones presented in **Table 1**.

From the 21 differently started analyses, we selected the one with lowest stress. The resulting normalized raw stress values for the analyses of the E-SVS variables and the E-PVQ variables, respectively, were 0.027, and 0.029, and the associated DAF’s were 0.973 and 0.971. The resulting configurations are plotted in **Figure 1**. Clearly, in both solutions four clusters can be discerned, but it can also be seen that one of the egoistic items is located close to the cluster of hedonic items. This is the egoistic item “ambitious,” which also in the OGM turned out to be strongly related to hedonic values. For the remainder, items of the subscales nicely form the theorized clusters.

#### Reliability of E-SVS and E-PVQ

No large differences were observed between the E-SVS and E-PVQ on the Cronbach’s alphas of the values scales (for E-SVS ranging from 0.63 to 0.88, for E-PVQ ranging from 0.68 to 0.88). In line with previous research and theorizing (Steg et al., 2014), when looking at each methodology separately, scales measuring the self-transcending biospheric and altruistic values were generally positively correlated (E-SVS: *r* = 0.41, E-PVQ: *r* = 0.27), and scales measuring the self-enhancing hedonic and egoistic values were also generally positively correlated (E-SVS: *r* = 0.35, E-PVQ: *r* = 0.29).

#### Correlations Between E-SVS and E-PVQ Scales

The E-PVQ and E-SVS scales correlated strongly with each other and all items from the E-PVQ correlated positively with the corresponding items from the E-SVS (see **Table 2**, last column), although sometimes these correlations were somewhat low (e.g., for “influential” it was only 0.31). When scrutinizing the correlation table for explorative purposes, we observed that E-SVS’s self-indulgent and E-PVQ’s enjoying life correlated 0.67 with each other, which was higher than with their corresponding items (respectively, *r* = 0.49 and *r* = 0.36). Yet, such observations should be treated with caution given the large number of correlations compared, the small sample size and accompanying large confidence intervals.

#### Relationships of E-PVQ and E-SVS With Environmental Self-Identity and Pro-environmental Beliefs and Behaviors

We correlated the scales from the E-PVQ and E-SVS with environmental self-identity and energy behaviors. As the purpose of this study was to compare the E-PVQ and E-SVS relationships with environmental self-identity and pro-environmental beliefs and behaviors – rather than showing which value (i.e., biospheric, altruistic, hedonic or egoistic) predicts the outcome variables best – we calculated the bivariate correlations between each value scale and each outcome variable (see **Table 3**).

**Table 3:** Bivariate correlations for each value, separated for E-SVS and E-PVQ, on each outcome variable.

	Biospheric values		Altruistic values		Hedonic values		Egoistic values	
	E-SVS	E-PVQ	E-SVS	E-PVQ	E-SVS	E-PVQ	E-SVS	E-PVQ
Environmental self-identity	0.61*	0.75*	0.20	0.26*	-0.21	-0.16	-0.04	-0.07
Energy wasting								
Leaving lights on when leaving the room	0.09	-0.02	0.01	-0.16	-0.01	-0.09	0.06	-0.07
Leaving appliances on stand-by	-0.23	-0.29*	-0.05	-0.17	0.35**	0.23	0.11	-0.15
Not fully loaded washing machine	-0.43*	-0.30*	-0.02	0.05	-0.01	0.00	0.03	0.12
Boiling more water than necessary	-0.16	-0.24*	-0.03	-0.22	0.30*	0.33**	0.05	0.00
Energy saving								
Doing laundry on cold temperatures	-0.01	-0.04	-0.03	0.01	-0.19	-0.14	0.03	0.06
Lowering thermostat when leaving home	0.15	0.26*	0.05	0.24*	-0.09	-0.15	0.19	0.18
Lowering thermostat when going to bed	0.11	0.10	0.11	0.18	-0.15	-0.13	0.12	0.10
Lowering thermostat 30 min before	0.15	0.13	0.08	0.16	-0.27*	-0.40*	0.13	0.10
Average thermostat temperature	-0.21	0.13	-0.15	-0.00	0.04	0.12	-0.06	0.03
Number of showers and baths a week	0.27*	0.03	0.13	0.02	0.13	0.02	0.35*	0.22
Duration of shower	-0.26*	-0.09	0.12	0.05	0.03	-0.13	0.01	-0.08

*Correlation coefficients larger than 0.24 had one-sided p-values below 0.05 and were marked with an asterisk. However, considering the large number of correlation coefficients inspected, single correlation coefficients should be interpreted cautiously.*

Despite some small differences between the E-PVQ and E-SVS, the correlations between the scales and the outcome variables were comparable, with no consistent pattern visible indicating that one scale correlated more strongly with environmental self-identity and energy behaviors than the other. In line with previous research, we found for both the E-SVS and E-PVQ a relatively strong correlation between biospheric values and environmental self-identity. The correlations between biospheric values and energy behaviors were mostly in the expected direction, but much smaller, and mostly not statistically significant. Altruistic, hedonic and egoistic values did not clearly relate to environmental self-identity and energy behaviors.

### Discussion

In Study 1 we tested a methodology – the E-PVQ – for measuring four basic human values that relate to environmental identity, beliefs and behaviors. The results of Study 1 supported the validity and reliability of the E-PVQ. More specifically, results to a large extent confirmed the theorized factor structure that distinguishes between biospheric, altruistic, biospheric, and hedonic values, and showed fair to good internal consistency for each value scale. Moreover, respondents rated the E-PVQ scale introduction, as well as the individual items, as clear and easy to understand.

Besides, the E-PVQ value clusters strongly related to the corresponding E-SVS value clusters, and most E-PVQ items correlated strongly with the corresponding item of the E-SVS. Nonetheless, for some items these relationships were smaller than we would have expected beforehand (e.g., enjoying life, being influential). Although it is quite likely that such exceptions are caused by chance (given the large number of correlation coefficients we inspected), one alternative explanation could be that the indirect way of asking about someone’s values in the E-PVQ made respondents less concerned about self-presentation than when answering the E-SVS (Schwartz, 2003) and therefore answered hedonic and egoistic items differently. In addition, some items (e.g., peace and ambitious) were relatively strongly related to another value cluster (respectively, biospheric and hedonic values) within the same value dimension (respectively, self-transcendence and self-enhancement). For the E-SVS, these correlations even exceeded the corrected correlation with the scale to which the items belonged, suggesting that a slightly different value structure might have fitted the data better. Nonetheless, some overlap within clusters from the same dimension is not surprising since they are theoretically related (Schwartz, 2012; Schwartz et al., 2012).

When compared, the E-PVQ performed equally well or even slightly better than the E-SVS. More specifically, most respondents indicated they preferred the E-PVQ over the E-SVS on ease and comprehensibility, which is in line with previous comparisons between the original SVS and PVQ (Schmidt et al., 2007; Schwartz et al., 2012). These findings are promising in support of the E-PVQ’s (and E-SVS’s) reliability, validity and usability. However, the results are based on a small sample. Accordingly, it is important to replicate these findings in a larger sample, preferably consisting of another group of people. For this reason, as well as to test the E-PVQ in a sample consisting of people who are not used to this kind of questions (Schwartz, 2003; Schwartz et al., 2012), we conducted a second study among a larger sample of secondary school students.

## Study 2

### Materials and Methods

Similar to Study 1, the goal of Study 2 was to validate the new E-PVQ and compare it with the E-SVS by looking at their factor structure, their reliability, their correlation with each other and their correlation with environmental beliefs and behaviors. More specifically, we wanted to replicate Study 1 with a larger sample consisting of secondary school students who were not used to answering these kind of questionnaires.

#### Participants

A paper-and-pencil questionnaire was conducted in 2017. Participants (*n* = 155, 77% female, 33% male) were Dutch secondary school students (age 14–18, *M*_age_ = 15.67, *SD* = 0.68) who voluntarily signed-up for an introductory practicum to learn about and experience what it means to do research. The study^6^ was part of this seminar. After participation, students were informed about the purpose of the study and its potential implications. In addition, students were asked to share their observations and ideas about the study, which were further discussed in class. Since we used a paper-and-pencil questionnaire, in which we could not remind the respondent that they missed a question, we had some missing data throughout the questionnaire. We excluded these in the relevant analyses.

#### Procedure and Measures

After agreeing on informed consent, participants could start the paper-and-pencil questionnaire. Participants were first asked for their gender, city of residence and birth year, after which they received either the E-SVS (*n* = 71) or E-PVQ (*n* = 84), which were identical to the ones used in Study 1 (for descriptive statistics, see **Table 4**). Thereafter, we presented questions (all measured on a 7-point Likert scale) about respondents’ ideas on the effect of climate change on the Netherlands (1 *very negative* to 7 *very positive*, *M* = 2.86, *SD* = 2.56), whether they thought climate change was something negative or positive (1 very *negative* to 7 very *positive*, *M* = 2.56, *SD* = 1.05), their pro-environmental personal norms (2 items from Steg and De Groot, 2010: I feel guilty when not acting environmentally friendly, I feel proud when acting environmentally friendly, 1 *totally not* to 7 *totally*; *r* = 0.51, *M* = 4.25, *SD* = 1.39), whether they were willing to save energy (1 *totally not* to 7 *totally*, *M* = 4.31, *SD* = 1.32), whether they were saving energy (1 *totally not* to 7 *totally*, *M* = 3.23, *SD* = 1.49), whether they thought climate change was a relevant topic (1 *totally irrelevant* to 7 *totally relevant*, *M* = 5.22, *SD* = 1.29) and how much they thought the government should invest in the environment (2 items, “nature preservation” and “reducing environmental problems,” 1 *nothing at all* to 7 *a lot*; *r* = 0.56, *M* = 4.37, *SD* = 1.39), followed by the other value questionnaire (i.e., either E-PVQ or E-SVS, respectively). So, eventually, all individuals completed both scales.

**Table 4:** Corrected correlations between value items and value clusters for both E-SVS and E-PVQ via oblique multiple group method, and the correlation between the E-SVS and corresponding E-PVQ item.

	Value cluster								
	E-SVS				E-PVQ				
	Bio	Alt	Hed	Ego	Bio	Alt	Hed	Ego	*r*
**Biospheric**	**Cronbach’s alpha = 0.869**				**Cronbach’s alpha = 0.881**				**0.78**

Pollution	**0.77**	0.45	0.03	0.01	**0.76**	0.34	0.00	-0.05	0.62
Protection	**0.76**	0.33	-0.02	-0.02	**0.80**	0.32	0.04	-0.01	0.63
Respect	**0.74**	0.44	0.01	-0.03	**0.73**	0.41	0.05	-0.12	0.58
Unity	**0.72**	0.27	0.01	-0.01	**0.68**	0.26	-0.01	-0.03	0.75

**Altruistic**	**Cronbach’s alpha = 0.632**				**Cronbach’s alpha = 0.761**				**0.67**

Equal	0.34	**0.37**	-0.08	-0.08	0.25	**0.51**	-0.01	-0.12	0.53
Social justice	0.25	**0.56**	-0.01	0.07	0.29	**0.55**	0.18	-0.16	0.34
Taking care	–	**–**	–	–	0.29	**0.64**	0.15	-0.01	0.31^a^
Peace	0.37	**0.31**	0.05	-0.07	0.33	**0.40**	0.03	-0.05	0.63
Helpful	0.26	**0.43**	0.04	0.06	0.23	**0.60**	0.14	0.02	0.60

**Hedonic**	**Cronbach’s alpha = 0.826**				**Cronbach’s alpha = 0.665**				**0.66**

Pleasure/fun	0.01	0.01	**0.66**	0.43	0.03	0.03	**0.57**	0.28	0.52
Enjoying life	0.03	0.03	**0.72**	0.32	0.16	0.14	**0.45**	0.26	0.47
Self-indulgent	0.01	0.00	**0.67**	0.44	-0.16	0.13	**0.43**	0.23	0.51

**Egoistic**	**Cronbach’s alpha = 0.695**				**Cronbach’s alpha = 0.694**				**0.65**

Social power	0.05	-0.02	0.16	**0.57**	0.00	-0.05	0.16	**0.58**	0.56
Authority	0.01	0.12	0.22	**0.53**	-0.09	-0.11	0.12	**0.64**	0.42
Influential	0.07	0.00	0.21	**0.47**	0.03	-0.02	0.16	**0.55**	0.52
Wealth	-0.14	-0.20	0.44	**0.37**	-0.12	-0.20	0.35	**0.34**	0.74
Ambitious	-0.02	0.07	0.46	**0.32**	-0.04	0.14	0.36	**0.15**	0.69

*M*	3.19	4.85	5.00	2.42	3.94	5.56	6.08	3.70	
*SD*^b^	1.46	1.07	1.35	1.09	1.25	0.83	0.75	0.94	

*Boldfaced correlation coefficients indicate the corrected item-total correlations between an item and the value cluster to which the item belongs. Correlation coefficients larger than 0.13 had one-sided p-values below 0.05. ^a^E-SVS’s item measuring social justice also contained the taking care component, which is why we correlated the E-PVQ’s taking care item with the E-SVS’s social justice item. ^b^We report untransformed SDs. For direct comparisons, one should adjust for the measuring scale used, which could be done by dividing the SDs of the E-SVS by 9 and multiplying them by 7. This results in SDs of 1.13, 0.83, 1.05, and 0.85, respectively, which are comparable to the SDs observed for the E-PVQ*.

### Results

We ran the same analyses as in Study 1, namely OMG method type of confirmatory factor analysis (e.g., Guttman, 1952; Nunnally, 1978; Stuive, 2007), MDS, reliability analyses, correlation analyses between value scales within each methodology, correlational analyses between value items and scales from each methodology, and correlational analyses between each value scale and the outcome variables.

#### Verifying Value Clusters

**Table 4** shows that for the E-SVS all but three items correlated strongest with the value scale with which it was supposed to be associated. The item “a world at peace” correlated most with the biospheric value scale (and not its own altruistic scale). Similarly, the items “ambitious” and “wealth” correlated most strongly with the hedonic value scale (rather than with the egoistic scale). For the E-PVQ (see **Table 4**), all but two items correlated strongest with the value scale to which the item theoretically belonged. The items measuring ambition and wealth correlated most strongly with the hedonic value scale, rather than with the egoistic scale.

#### Multidimensional Scaling

**FIGURE 2:**
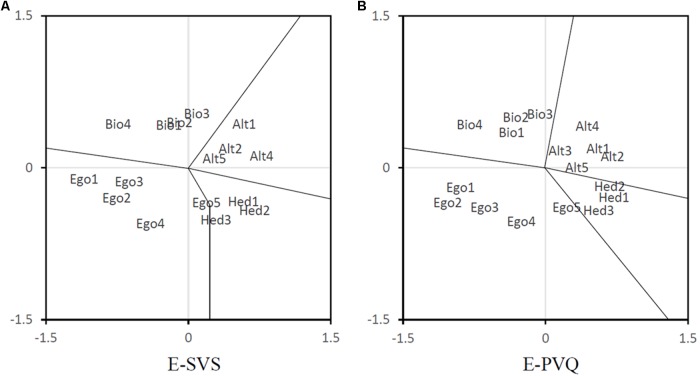
Two-dimensional multidimensional scaling for the **(A)** E-SVS and **(B)** E-PVQ tested in Study 2. Item abbreviations (e.g., Bio1) correspond to the ones presented in **Table 1**.

To further inspect how the value items of the E-SVS and E-PVQ cluster together, we performed the same MDS as we did for Study 1. From the 21 differently started analyses, we selected the one with lowest stress. The resulting normalized raw stress values were 0.030 for the E-SVS analysis and 0.026 for the E-PVQ analysis, the associated DAF’s were 0.970 and 0.974, respectively. The resulting configurations are plotted in **Figure 2**. Again, in both solutions the four value clusters can be clearly discerned, except for the egoistic item “ambitious,” which was located close to the hedonic cluster.

#### Reliability of E-SVS and E-PVQ

Like Study 1, both scales (fairly) reliably measured each of the value types (Cronbach’s alphas varied from 0.63 to 0.88), without consistent difference across the two questionnaires. Inspection of the correlations between the value scales within each methodology indicated that biospheric and altruistic values were generally positively correlated with each other (E-PVQ: *r* = 0.38; E-SVS: *r* = 0.44), and hedonic and egoistic values were also generally positively correlated (E-PVQ: *r* = 0.33; E-SVS: *r* = 0.44), which is in line with previous theorizing (Schwartz, 2012; Steg et al., 2014).

#### Correlation Between E-SVS and E-PVQ Scales

The E-PVQ and E-SVS scales correlated strongly with each other and all items from the E-PVQ correlated positively with the corresponding items from the E-SVS (see **Table 4**, last column). Only the E-PVQ “social justice” and “taking care” items were weakly related to the corresponding E-SVS item (which included both elements).

#### Relationships of E-PVQ and E-SVS With Pro-environmental Beliefs and Behaviors

We first inspected the correlations of the biospheric value scale measured with the E-PVQ and E-SVS with all outcome variables (see **Table 5**). Although slight differences between the scales could be observed, there was no clear pattern indicating that one scale related more strongly to the outcome variables than the other. For both scales, biospheric values correlated with all outcome variables in the expected direction. That is, higher biospheric values were indicative for stronger climate change beliefs, pro-environmental personal norms, willingness and engagement in energy saving behaviors and support for sustainable governmental investments. Similar, though smaller, correlations could be observed for altruistic values. Correlations for hedonic and egoistic values were in general small (absolute values smaller than 0.20) and, accordingly, difficult to interpret.

**Table 5:** Bivariate correlations for each value, separated for E-SVS and E-PVQ, on each outcome variable of Study 2.

	Biospheric values		Altruistic values		Hedonic values		Egoistic values	
	E-SVS	E-PVQ	E-SVS	E-PVQ	E-SVS	E-PVQ	E-SVS	E-PVQ
Effect of climate change on NL	-0.25^∗^	-0.30^∗^	-0.08	-0.03	-0.01	0.02	-0.07	-0.02
Attitudes toward climate change	-0.25^∗^	-0.37^∗^	-0.13	-0.14	-0.05	-0.06	0.12	0.03
Relevance of climate change	0.51^∗^	0.54^∗^	0.29^∗^	0.25^∗^	-0.08	0.03	-0.07	-0.03
Personal norms	0.61^∗^	0.57^∗^	0.32^∗^	0.32^∗^	-0.16^∗^	-0.03	-0.17^∗^	-0.13
Willingness to save energy	0.46^∗^	0.42^∗^	0.32^∗^	0.20^∗^	-0.09	-0.01	-0.16^∗^	-0.06
Energy saving behavior	0.34^∗^	0.39^∗^	0.22^∗^	0.20^∗^	-0.19^∗^	-0.13	-0.11	0.01
Investing in environment	0.70^∗^	0.62^∗^	0.33^∗^	0.23^∗^	-0.09	0.02	-0.04	-0.03

*Correlation coefficients larger than 0.14 had one-sided p-values below 0.05 and were marked with an asterisk. However, considering the large number of correlation coefficients inspected, single correlation coefficients should be interpreted cautiously.*

### Discussion

Study 2 again supported the reliability and validity of both the E-PVQ and E-SVS, in another population than Study 1. Similar to the results of Study 1, the theorized value structure was again to a large extent confirmed for both scales and the internal consistency was good for each single value cluster. However, for both the E-SVS and the E-PVQ, the “wealth” and “ambitious” items were only weakly related to the egoistic value scale to which they belong theoretically, and related similarly to the hedonic value scale. That both items relate to both the egoistic and hedonic value is not surprising since both values are related to the self-enhancement dimension (Schwartz, 2012; Schwartz et al., 2012). Nevertheless, the weak correlation with its own value cluster deserves to be explored in more detail in future research.

The E-PVQ value scales related strongly to the E-SVS value scales, and most items measured with the E-PVQ related strongly with the corresponding item from E-SVS. However, the E-PVQ items “social justice” and “taking care” were only weakly related to corresponding single item from the E-SVS that measured both constructs together. This weak relationship could be caused by the E-SVS item asking about two separate constructs; more research is needed to test this.

Both the E-PVQ and E-SVS were similarly related to environmental outcome variables. More specifically, this relationship was most prevalent for biospheric values, which related to all environmental outcome variables in the expected directions, replicating earlier research (Stern et al., 1998; Schultz and Zelezny, 1999; Stern, 2000; Nordlund and Garvill, 2002, 2003; De Groot and Steg, 2007, 2008, 2009; Hansla et al., 2008; Boer and Fischer, 2013; Steg et al., 2014; Nilsson et al., 2016). That is, the stronger respondents endorsed biospheric values, the more they were concerned with the environment and climate change, and the more likely they were to think and act pro-environmentally. Similarly, most of these relationships were also, although weaker, observable for altruistic values, but were more ambiguous and inconclusive for hedonic and egoistic values.

## General Discussion

In two studies, collected among two different population samples, we clearly and consistently showed the validity, reliability, and usability of the E-SVS and the E-PVQ, representing two different ways of measuring human values that are expected to relate to environmental self-identity, environmental beliefs, and behaviors. In general, both methodologies to a large extent confirmed the theorized factor structure, which differentiates between biospheric, altruistic, egoistic, and hedonic values (Stern et al., 1998; De Groot and Steg, 2008; Steg et al., 2014). The two studies support each other by indicating virtually the same structure in the items. For both the E-SVS and E-PVQ, biospheric and altruistic values related to each other (i.e., the self-transcendence dimension) and egoistic and hedonic values related to each other (i.e., the self-enhancement dimension), which is in line with previous theorizing and findings (Stern et al., 1998; Abrahamse et al., 2007; De Groot and Steg, 2008; Steg et al., 2014) and supports the measures’ convergent validity. Moreover, values from the self-transcendence cluster were only weakly related to values of the self-enhancement cluster, which is also in line with previous theorizing and results (Schwartz, 1992, 2012; Stern et al., 1998; De Groot and Steg, 2008; Steg et al., 2014) and supports the measures’ discriminant validity. In addition, values measured following both methodologies strongly related to each other, and related comparably with the outcome variables, which supports the scales’ concurrent and construct validity.

Whereas both methodologies seem suitable for measuring the human values related to environmental beliefs and behaviors, we also observed some differences in outcomes between the two methodologies. In line with previous comparisons between the SVS and PVQ methodologies (Schwartz, 2003, 2005; Schmidt et al., 2007; Schwartz et al., 2012; Schwartz and Butenko, 2014), respondents seemed to slightly prefer the E-PVQ over the E-SVS on ease of use and comprehensibility (Study 1). Accordingly, and as argued by Schwartz (2003), it seems like asking respondents to rate the similarity of another person to oneself is less cognitively demanding for most respondents than asking them to directly rate abstract values. Considering that the value questionnaire consists of relatively many items and requires quite some effort from respondents, it is important to take factors like ease-of-use, comprehensibility and clarity into account when deciding on which methodology to use – making the E-PVQ a slightly better choice.

That said, it is important to emphasize that the E-SVS was performing comparably well overall and that in specific situations the E-SVS could be more suitable. For instance, because the E-SVS is commonly used within the field of environmental psychology (Stern et al., 1998; Schwartz, 2003; Steg et al., 2005; De Groot and Steg, 2007; Steg and De Groot, 2012; Steg et al., 2014), the E-SVS would be the choice of preference when a study aims to make direct comparisons with data collected in the past. Alternatively, since the E-PVQ requires gender-matching (i.e., the formulation of the items is adjusted to the gender of the respondent), the E-SVS is more suitable for data collection in situations where you cannot take someone’s gender into account while administering the value questions. This could for instance be the case when sending out paper-and-pencil questionnaires to households without having knowledge about who in the household will fill it out. Hence, both the E-PVQ and E-SVS perform well, and both could be more suitable for measuring human values related to environmental behaviors and beliefs in specific situations.

Our studies also raise some new questions that could be addressed in future studies^7^. Firstly, it is important to note that the E-SVS and E-PVQ methodologies differ in their formulation of the items as well as the scale they used, which makes it difficult to identify what exactly made respondents slightly prefer the E-PVQ over the E-SVS. For instance, the preference for the E-SVS might be caused by the symmetrical scale it employs rather than the wording of the items, leaving the question open what would happen when a more symmetrical scale was used for the E-SVS (see also Stern et al., 1998). Although this could be an interesting research question in itself, it should be noted that the use of the non-symmetrical scale for the E-SVS is common practice and considered necessary to warrant enough variance between the items (Schwartz, 1992, 1994, 2003). The use of a non-symmetrical scale for the E-SVS could therefore be seen as an implication of the wording of the items, rather than a choice of the researcher.

Secondly, although we tested the E-SVS and E-PVQ in two rather distinct samples, further research is needed to test the suitability of both measures in other samples. It might be specifically interesting to further test the E-PVQ and compare it with the E-SVS in populations which are known to have difficulties with the E-SVS, such as children under 14, elderly and people from non-Western countries (Schwartz et al., 2001, 2012; Schwartz, 2003; Schwartz and Butenko, 2014). Accordingly, based on previous theorizing (Schwartz et al., 2001, 2012; Schwartz, 2003; Schwartz and Butenko, 2014) and our initial findings regarding the ease of use and comprehensibility of the E-PVQ, one would hypothesize to observe stronger preference for the E-PVQ in samples who are known to have difficulties with the E-SVS; future research is needed to test this hypothesis. That said, we believe that our samples are appropriate for the current paper’s goal; that is, testing whether the E-PVQ is a viable alternative to the E-SVS. In fact, our comparisons could be considered a conservative test since the E-SVS is well-validated and often used among similar samples as ours.

Furthermore, more research could be done on the relationship between self-enhancing egoistic and hedonic values and environmental beliefs and behavior. Whereas the self-transcending biospheric and altruistic values were clearly related to the environmental beliefs and behaviors we measured, the self-enhancing egoistic and hedonic values only inconsistently and, at best, weakly related to these behaviors. Previous studies also have shown that the relationship between self-enhancement and environmental beliefs and behaviors is weaker and more instable than for self-transcendence values, and strongly depends on the hedonic and egoistic costs of the beliefs and behavior at stake (Stern et al., 1998; De Groot and Steg, 2008; Steg et al., 2015, 2014). Future research could test relationships between values and a wider range of beliefs and behaviors, including beliefs and behaviors with low and high hedonic and egoistic costs (and benefits), to examine whether such beliefs and behaviors would be more strongly related to hedonic and egoistic values.

In sum, our findings give a consistent picture of the validity and reliability of the E-PVQ and the commonly employed E-SVS (Stern et al., 1998; De Groot and Steg, 2008; Steg et al., 2015, 2014). Based on two studies, both methodologies seem suitable for measuring human values and their relationships with environmental beliefs and behaviors. Yet, the results indicate that the E-PVQ is slightly easier to complete and more comprehensible for respondents.

## Ethics Statement

This study was carried out in accordance with the recommendations of ‘Law on Medical Research involving Human Beings (WMO), the professional code of the NIP, and the Personal Data Protection Act (WBP), and approved by the Ethical Committee Psychology of the University of Groningen’ with written informed consent from all subjects in accordance with the Declaration of Helsinki.

## Author Contributions

TB and LS developed the study concept and contributed to the analysis interpretation. TB, LS, and HK contributed to the study design and implementation. TB collected the data, performed the data analyses, and drafted the manuscript. LS and HK provided critical revisions. All authors approved the final version of the manuscript for submission.

## Conflict of Interest Statement

The authors declare that the research was conducted in the absence of any commercial or financial relationships that could be construed as a potential conflict of interest.

## Footnotes

1 The brief E-SVS only includes items from the self-transcendence and self-enhancement values that most consistently relate to environmental beliefs and behaviors.

2 This is in line with the principles of compatibility (Ajzen and Fishbein, 1977; Ajzen, 2005) that proposes that attitude-behavior relations are only found when elements, such as target and action, strongly overlap with each other.

3 Based on comments from a pilot, we decided to split the altruistic E-SVS item “SOCIAL JUSTICE (correcting injustice, care for the weak)” in two E-PVQ items “it is important to [him/her] to take care of those who are worse off” and “… that every person is treated justly.” We included both items in the E-PVQ.

4 For explorative reasons we included two additional egoistic items focusing on status and getting recognition, since these topics seemed underrepresented in the E-SVS. The inclusion of these items in the egoistic scale did not affect any of our conclusions; therefore, we decided to report the analyses of only those items of the E-PVQ that parallel the items of the E-SVS.

5 Although Schwartz typically employs a 6-point scale (Schwartz, 2003; Schwartz et al., 2012), we decided to work with a 7-point scale to give respondents the opportunity to differentiate more accurately between the items.

6 Study 2 was part of a larger study including questions about media, politics and the refugee crisis, as the current paper focuses exclusively on the measurement of personal values and the correlation of personal values with environmental attitudes and beliefs and behaviors, we decided to only report those variables which were relevant for purpose.

7 One of the reviewers raised the concern that relationships between scales could be prone to shared-method variance and semantic overlap between scales (e.g., McBroom and Reed, 1992; Arnulf et al., 2014). Yet, we do not think these issues significantly impacted our conclusions for at least three reasons. Firstly, many of the outcome variables were measured in a different way than the E-SVS or E-PVQ. For instance, outcome variables of Study 1 focused on concrete behaviors (e.g., thermostat settings, boiling water), and used other scales than were used for the values (i.e., low shared-method variance) and did not use language directly referring to nature or the environment (i.e., low semantic overlap). Secondly, the goal of the current paper was to compare the performance of the E-PVQ and E-SVS (see also Schwartz and Butenko, 2014), and we assume these issues would have impacted findings for both scales in comparable ways. If anything, we expect these potential biases would favor the E-SVS since this measure is more comparable to the other scales than the indirectly formulated E-PVQ, which is not what we observed in our results. Thirdly, any semantic overlap between variables of interest is likely to be caused by the theorized relationship between the variables. For instance, the purchase of Granny Smith apples is more likely to be predicted by liking of apples than liking of pears, not because one uses the same word but because the action (purchase) and attitude (liking) have similar targets, for which – quite obviously – similar words are used. The same reasoning applies for the relationship between environmental behaviors (purchase of Granny Smith apples) and biospheric (liking of apples) and altruistic values (liking of pears).
