# Supplementary material for: Measuring Values in Environmental Research: A Test of an Environmental Portrait Value Questionnaire
Source: Front Psychol. 2018 Apr 23;9:564. doi: 10.3389/fpsyg.2018.00564 (PMC5931026; doi:10.3389/fpsyg.2018.00564)
Supplement: Supplementary file 1 [file Table_1.docx]

| Table 1. *Items for measuring the values “universalism” (Schwartz, 1992), “universalism-nature” (Schwartz et al., 2012, 2016) and “biospheric” values (Steg et al., 2014, E-PVQ)* | | | |
| --- | --- | --- | --- |
| SVS,  Schwartz, 1992; Steg et al., 2014 | PVQ5X - PVQ-R,  Schwartz et al., 2012 | PVQ-RR,  Schwartz, 2016 | E-PVQ |
| PREVENTING POLLUTION (protecting natural resources)* | Protecting the natural environment from destruction or pollution is important to [him/her]. | It is important to [him/her] to protect the natural environment from destruction or pollution. | It is important to [him/her] to prevent environmental pollution. |
| PROTECTING THE ENVIRONMENT (preserving nature) | It is important to [him/her] to work against threats to the world of nature. | It is important to [him/her] to take part in activities to defend nature. | It is important to [him/her] to protect the environment. |
| RESPECTING THE EARTH (harmony with other species)* | ---------------------------- | ---------------------------- | It is important to [him/her] to respect nature. |
| UNITY WITH NATURE (fitting into nature) | ---------------------------- | ---------------------------- | It is important to [him/her] to be in unity with nature. |
| ---------------------------- | [He/She] strongly believes that [he/she] should care for nature. | It is important to [him/her] to care for nature. | ---------------------------- |
| *Note.* An asterisk denotes an item that was later (De Groot & Steg, 2008; Stern et al., 1998) added to the original SVS (Schwartz, 1992). | | | |
