# Supplementary material for: Measuring Values in Environmental Research: A Test of an Environmental Portrait Value Questionnaire
Source: Front Psychol. 2018 Apr 23;9:564. doi: 10.3389/fpsyg.2018.00564 (PMC5931026; doi:10.3389/fpsyg.2018.00564)
Supplement: Supplementary file 2 [file Table_2.docx]

| Table 2. *Items for measuring the values “universalism”, “benevolence” (Schwartz, 1992), “universalism-concern”, “universalism-tolerance”, “benevolence-care” (Schwartz et al., 2012, 2016) and “altruistic” values (Steg et al., 2014, E-PVQ)* | | | |
| --- | --- | --- | --- |
| SVS,  Schwartz, 1992; Steg et al., 2014 | PVQ5X - PVQ-R,  Schwartz et al., 2012 | PVQ-RR,  Schwartz, 2016 | E-PVQ |
| EQUALITY (equal opportunity for all) | [He/She] thinks it is important that every person in the world have equal opportunities in life. | It is important to [him/her] that every person in the world have equal opportunities in life. | It is important to [him/her] that every person has equal opportunities. |
| SOCIAL JUSTICE (correcting injustice, care for the weak) | Protecting society’s weak and vulnerable members is important to [him/her]. | It is important to [him/her] that the weak and vulnerable in society be protected. | It is important to [him/her] to take care of those who are worse off. |
| ---------------------------- | [He/She] wants everyone to be treated justly, even people [he/she] doesn’t know. | It is important to [him/her] that everyone be treated justly, even people [he/she] doesn’t know. | It is important to [him/her] that every person is treated justly. |
| A WORLD AT PEACE (free of war and conflict) | [He/she] works to promote harmony and peace among diverse groups.* | It is important to [him/her] to be tolerant toward all kinds of people and groups. | It is important to [him/her] that there is no war or conflict. |
| ---------------------------- | It is important to [him/her] to listen to people who are different from [him/her]. | It is important to [him/her] to listen to and understand people who are different from [him/her]. | ---------------------------- |
| ---------------------------- | Even when [he/she] disagrees with people, it is important to [him/her] to understand them. | It is important to [him/her] to accept people even when [he/she] disagrees with them. | ---------------------------- |
| HELPFUL (working for the welfare of others) | It’s very important to [him/her] to help the people dear to [him/her]. | It is very important to [him/her] to help the people dear to [him/her]. | It is important to [him/her] to be helpful to others. |
| ---------------------------- | Caring for the well-being of people [he/she] is close to is important to [him/her]. | It is important to [him/her] to take care of people [he/she] is close to. | ---------------------------- |
| ---------------------------- | [He/She] tries always to be responsive to the needs of [his/her] family and friends.* | It is important to [him/her] to concern [himself/herself] with every need of [his/her] dear ones. | ---------------------------- |
| *Note.* An asterisk denotes an item that did not fit the model (Schwartz et al., 2012), Schwartz and colleagues (2012) suggest to replace these items by a revision, which overlaps with the corresponding item in the PVQ-RR (Schwartz, 2016) | | | |
