# Supplementary material for: Measuring Values in Environmental Research: A Test of an Environmental Portrait Value Questionnaire
Source: Front Psychol. 2018 Apr 23;9:564. doi: 10.3389/fpsyg.2018.00564 (PMC5931026; doi:10.3389/fpsyg.2018.00564)
Supplement: Supplementary file 3 [file Table_3.docx]

| Table 3. *Items for measuring the values “hedonism” (Schwartz, 1992; Schwartz et al., 2012, 2016) and “hedonic” values (Steg et al., 2014, E-PVQ)* | | | |
| --- | --- | --- | --- |
| SVS,  Schwartz, 1992; Steg et al., 2014 | PVQ5X - PVQ-R,  Schwartz et al., 2012 | PVQ-RR,  Schwartz, 2016 | E-PVQ |
| SELF-INDULGENT (doing pleasant things) | [He/She] takes advantage of every opportunity to have fun.* | It is important to [him/her] to take advantage of every opportunity to have fun. | It is important to [him/her] to have fun. |
| ENJOYING LIFE (enjoying food, sex, leisure, etc.) | Enjoying life’s pleasures is important to [him/her]. | It is important to [him/her] to enjoy life’s pleasures. | It is important to [him/her] to enjoy the life’s pleasures. |
| PLEASURE (gratification of desires) | Having a good time is important to [him/her]. | It is important to [him/her] to have a good time. | It is important to [him/her] to do things [he/she] enjoys. |
| *Note.* An asterisk denotes an item that did not fit the model (Schwartz et al., 2012), Schwartz and colleagues (2012) suggest to replace these items by a revision, which overlaps with the corresponding item in the PVQ-RR (Schwartz, 2016) | | | |
