# Supplementary material for: Measuring Values in Environmental Research: A Test of an Environmental Portrait Value Questionnaire
Source: Front Psychol. 2018 Apr 23;9:564. doi: 10.3389/fpsyg.2018.00564 (PMC5931026; doi:10.3389/fpsyg.2018.00564)
Supplement: Supplementary file 4 [file Table_4.docx]

| Table 4. *Items for measuring the values “power” (Schwartz, 1992), “power-dominance”, “power-resources” (Schwartz et al., 2012, 2016), “achievement” (Schwartz, 1992; Schwartz et al., 2012, 2016) and “egoistic” values (Steg et al., 2014, E-PVQ)* | | | |
| --- | --- | --- | --- |
| SVS,  Schwartz, 1992; Steg et al., 2014 | PVQ5X - PVQ-R,  Schwartz et al., 2012 | PVQ-RR,  Schwartz, 2016 | E-PVQ |
| SOCIAL POWER (control over others, dominance) | [He/she] wants people to do what [he/she] says. | It is important to [him/her] that people do what [he/she] says they should. | It is important to [him/her] to have control over others’ actions. |
| AUTHORITY (the right to lead or command) | It is important to [him/her] to be the one who tells others what to do. | It is important to [him/her] to be the one who tells others what to do. | It is important to [him/her] to have authority over others. |
| INFLUENTIAL (having an impact on people and events) | It is important to [him/her] to be the most influential person in any group. * | It is important to [him/her] to have the power to make people do what [he/she] wants | It is important to [him/her] to be influential. |
| WEALTH (material possessions, money) | Being wealthy is important to [him/her]. | It is important to [him/her] to be wealthy. | It is important to [him/her] to have money and possessions. |
| ---------------------------- | [He/She] pursues high status and power. * | It is important to [him/her] to own expensive things that show [his/her] wealth. | ---------------------------- |
| ---------------------------- | Having the feeling of power that money can bring is important to [him/her]. | It is important to [him/her] to have the power that money can bring. | ---------------------------- |
| AMBITIOUS (hardworking, aspiring) | Having ambitions in life is important to [him/her]. | It is important to [him/her] to have ambitions in life | It is important to [him/her] to work hard and be ambitious. |
| ---------------------------- | Being very successful is important to [him/her]. | It is important to [him/her] to be very successful. | ---------------------------- |
| ---------------------------- | It is important to [him/her] to have people admire what [he/she] achieves. | It is important to [him/her] that people recognize what [he/she] achieves. | ---------------------------- |
| *Note.* An asterisk denotes an item that did not fit the model (Schwartz et al., 2012), Schwartz and colleagues (2012) suggest to replace these items by a revision, which overlaps with the corresponding item in the PVQ-RR (Schwartz, 2016) | | | |
